# Floor Sensing System Using Laser Reflectivity for Localizing Everyday Objects and Robot

**DOI:** 10.3390/s140407524

**Published:** 2014-04-24

**Authors:** Yoonseok Pyo, Tsutomu Hasegawa, Tokuo Tsuji, Ryo Kurazume, Ken'ichi Morooka

**Affiliations:** 1 Graduate School of Information Science and Electrical Engineering, Kyushu University, 744 Motooka, Nishi-ku, Fukuoka-shi, Fukuoka 819-0395, Japan; 2 Kumamoto National College of Technology, Koshi-shi, Kumamoto 861-1102, Japan; E-Mail: hasegawa@kumamoto-nct.ac.jp; 3 Faculty of Information Science and Electrical Engineering, Kyushu University, 744 Motooka, Nishi-ku, Fukuoka-shi, Fukuoka 819-0395, Japan; E-Mails: tsuji@ait.kyushu-u.ac.jp (T.T.); kurazume@ait.kyushu-u.ac.jp (R.K.); morooka@ait.kyushu-u.ac.jp (K.M.)

**Keywords:** laser range finder, position measurement, service robots

## Abstract

This paper describes a new method of measuring the position of everyday objects and a robot on the floor using distance and reflectance acquired by laser range finder (LRF). The information obtained by this method is important for a service robot working in a human daily life environment. Our method uses only one LRF together with a mirror installed on the wall. Moreover, since the area of sensing is limited to a LRF scanning plane parallel to the floor and just a few centimeters above the floor, the scanning covers the whole room with minimal invasion of privacy of a resident, and occlusion problem is mitigated by using mirror. We use the reflection intensity and position information obtained from the target surface. Although it is not possible to identify all objects by additionally using reflection values, it would be easier to identify unknown objects if we can eliminate easily identifiable objects by reflectance. In addition, we propose a method for measuring the robot's pose using the tag which has the encoded reflection pattern optically identified by the LRF. Our experimental results validate the effectiveness of the proposed method.

## 1. Introduction

Daily life assistance is one of the most important applications of service robots in the near future. A service robot must have a function for recognizing its surroundings. However, it is very difficult to recognize an environment of human daily life since a real space keeps changing dynamically. There exist human walking and working around while the space is cluttered with furniture and everyday objects. For a robot, it is quite difficult to recognize its surroundings by only using sensors mounted on its body. Instead of relying only on the on-board sensors and computers to recognize its surrounding situation, an informationally structured environment using distributed sensors embedded in the environment is a promising approach to the issue [1–5].

It is still very difficult to directly measure displacement of objects and human by using distributed and embedded sensors due to the various constraints in the daily life environment. For example, vision sensors suffer from illumination change and occlusion. In addition, deployment of multiple cameras is needed to complement the incompatibility of range of view and the resolution of sensing. However, embedding so many cameras so that everything is visible would need enormous manpower and cost due to initial setups and later maintenance of wiring, fixture, calibration, lighting control and so on. Furthermore it is not acceptable for residents in individual space like home, a patient room of hospital, nursing care facilities for elderly because of the possible invasion of their privacy: all their appearance including their body action, facial expression, clothes, and being naked in some cases are recorded day and night. The data may be stolen or peeped by malicious person.

In this paper, we propose a method to measuring position of everyday objects and a robot in a room, a private indoor space, while protecting privacy of residents. The method uses only one LRF fixed on the floor close to the wall, therefore the sensor implementation is very simple. Since the range of view is limited to a scanning plane parallel to the floor and just a few centimeters above the floor, the system causes minimal invasion of privacy while enabling acquisition of position data of human feet together with objects on the floor including small everyday objects. However, it is difficult to directly recognize the situation of surroundings since the available sensor data is limited. To overcome the difficulties, we introduce the following ideas.

First, we reduce the occlusion area by using a LRF and a mirror. The scanning plane of the LRF is carefully selected to be parallel and just a few centimeters above the floor so that there exist relatively few objects that cross the plane in our environment which contains a bed, chairs, tables and so on. This means that the sensing plane is less influenced by obstacles. In addition, a strip of mirror is attached to the side wall of the room so that the reflected laser scans the floor. The floor is scanned by the laser projected from two different positions. Therefore the furniture having legs with small cross sections will not cause severe occlusion. In the case of the furniture having large support on the floor, we can lift it using short and small pillars to create space below for the scanned laser to pass through thus reducing occlusion. This may be constraint but can be acceptable because of much bigger advantage of the floor sensing system: simple setting, low cost, low risk of privacy invasion, robust against illumination change.

Second, we identify the objects by using the reflection intensity and position information obtained from the target surface. The LRF provides both distance value and reflectance value when the laser beam is projected on the object surface. The reflectance value is a function of distance, angle of incidence of the laser to the surface, and optical property of the surface of the object. Therefore the reflectance value can be normalized in terms of the distance and the angle of incidence by using position data obtained by the LRF Objects can be distinguished by this normalized reflectance value if the difference is sufficiently large above the noise level.

Third, we use the retroreflective materials to improve detection and position measurement. Some objects have similar reflectance value. To expand the normalized reflectance value, a retroreflective tape is attached to the surface of the object of interest. The retroreflective material has a transparent surface in which micro glass balls are embedded so that the incident light is reflected back to the same direction, giving a very high reflection. Attaching the retroreflective tape on the supporting part of stable furniture and mobile objects like wheeled robots makes them distinguishable by its reflection value. Only a small tape is enough for the object, since the LRF scanning height from the bottom is always kept. Furthermore, we have developed a method for identifying the pose of objects by attaching a tag coded by reflection value. This method has been successfully applied to pose measurement of a cylindrical robot which has no geometrical features useful for pose identification.

The rest of the paper is organized as follows: after presenting related work in Section 2, we introduce the floor sensing system using a LRF and a mirror in Section 3. In Section 4 we describe the method of identification using laser reflectivity. Section 5 describes method for improvement of detection and position measurement by attaching retroreflective materials. Section 6 shows an experimental environment and experimental results. Section 7 concludes the paper.

## 2. Related Work

LRF has been extensively used on robot for various tasks, such as object detection/recognition, tracking moving targets. Moving object detection and tracking [6–15] are an important feature required by a robot, in particular for operations in dynamic environments. Detection of moving objects prior to any collision is necessary for safe navigation of a robot. The CMU-RI Navlab group [10,11] has developed such a detection and tracking of moving objects (DATMO) system that uses 2D and 3D laser scanner. The system is able to track on the order of 100 objects simultaneously. The applications that used DATMO included a collision warning system, pedestrian detection and classification, autonomous driving, human track learning and prediction. But, the target and environment of these application are significantly different from our case in this paper. Moreover, the general laser-based detection using only geometric information are not enough to identify objects in cluttered environments.

LRF has been used to measure human motion [16–19]. In most of the previous works, the laser scanning plane is set to be horizontal at the height of the waist of an adult. However, this configuration has some disadvantages: the position measurement is influenced by the motion of arms and hands; small children cannot be measured; and tall tables and chairs located at the central area of the room may cause occlusion. Therefore, instantaneous measurement may be less accurate and less reliable, even though the long term trend of motion is available. Pedestrian tracking is reported in [20] by setting the scanning plane at the height of human leg. Though it has successfully tracked pedestrians at the railway station in Tokyo, it cannot distinguish other objects existing within the measurement area.

Using cameras to track everyday objects in a room is reported in [21–24]. Also, objects detection methods using both laser and vision data was proposed [25–27]. Measurements using camera are influenced by change of illumination and occlusion. Furthermore it may not be desirable due to possible invasion of privacy in individual space. Everyday objects can be tracked once RFID or ultrasonic tags are attached to them [28,29]. However active scanning of large directional antennas is required for tracking RFID tags. This is time consuming and the resolution is rather low. In addition, the ultrasonic tag is too large and expensive to be used on numerous everyday objects.

Oishi *et al.* [30,31] proposed a colorization of 3D geometric model and smoothing of range image method using LRF reflectivity. Rusu *et al.* [32] proposed a perception to identify and annotate doors and handles using LRF reflectivity. On the one hand, we use the laser reflectivity for localizing everyday objects and robot. The laser reflectivity depends not only on the optical property of objects but also on the distance and the angle of incidence of the laser beam. For this reason, we consider the distance and the angle of incidence of the laser beam to identify people, robots, furniture and everyday objects.

In our daily life, we often leave or drop everyday objects on the floor. Also, various objects, such as people, other robots, or furniture may be on the floor. Therefore, detection and position measurement of these objects on the floor is an important issue in the informationally structured environment.

## 3. Floor Sensing Using Laser Range Finder and Mirror

We propose a method of position measurement using a LRF and a mirror. Many position measurement methods using a LRF have been proposed so far [16–18,20,33]. Multiple laser scanners have been exploited so that a relatively large area can be covered. The target of these methods are people or objects larger than a human in a rather large space. In contrast to this, our targets for position measurement are small everyday objects and robots in an indoor environment where multiple LRFs may not be installed due to space constraint and cost limitation. Therefore we use only one LRF and a strip of mirror attached to the side wall. We will show how this configuration is effective in measuring position of objects of various different optical properties while reducing occlusion.

**Figure 1.**
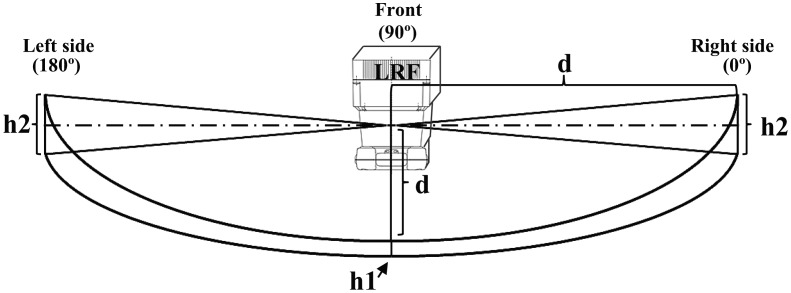
Diffuse feature of laser beam.

We installed the LRF(Hokuyo URG-30LX) at a height of about 2.7 centimeter above the floor to avoid unintentional reflection from the floor due to the diffusion of the laser beam (Figure 1, Table 1). A strip of mirror is attached to a side wall to reflect the laser beam from the LRF [34]. The whole area of both direct beam scanning from the LRF and indirect beam scanning via the mirror is a measurement region.

If no object is placed on the floor, the LRF measures the distance to the opposite wall. If an object is placed on the floor, the LRF measures the distance to the object. Even a small object is detected if it has a height of more than 2.7 centimeters. The LRF may not obtain any distance data due to the reflection property of the object. In this case, our system still obtains the distance value thanks to the combination of the LRF and the mirror as follows.

**Figure 2.**
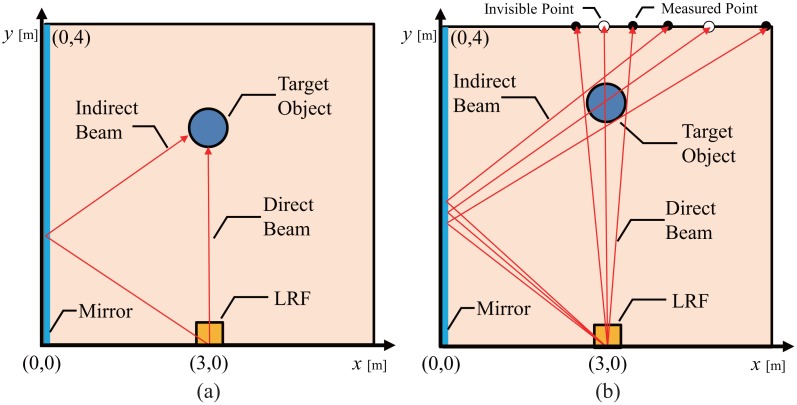
Position measurement using reflected laser beam and diffused laser beam: (**a**) position measurement using reflected laser beam, (**b**) position measurement using diffused laser beam.

(1) Position Measurement using Reflected Laser Beam: If an object reflects a sufficient laser beam, the LRF obtains the distance not to the wall but to the object. Then the system detects the existence of the object from this difference, and calculates the position using the distance and the angle of the laser beam reflected by the mirror (Figure 2a). Moreover, two types of the measurements can be obtained. One result is obtained using the direct laser beam from the object, and the other is obtained from the indirect laser beam via the mirror .
(2) Position Measurement using Diffused Laser Beam: If the placed object does not reflect the sufficient laser beam, e.g., a transparent plastic bottle, the LRF is unable to obtain any distance data. This implies that some object is placed somewhere on the line from the LRF to the wall. Also if the LRF fails to obtain indirect measurement, then some object is placed on the line from the LRF to the wall via the mirror. Dashed lines in Figure 2b show these two lines. By integrating these two pieces of information, we can calculate the position of the object as the intersection of the two lines.

The sensing performance of one LRF with a mirror may be improved by installing additional mirrors on other wall if there is not obstacle like a piece of large furniture in front of the wall. The occluded area by obstacles would be further reduced by a multi-reflected laser beam. In this case, limitation of the sensing is due to the diffusion of the laser beam of the LRF. Aperture of the beam grows as the distance is longer, then the laser is eventually reflected by the floor since the scanning plane is designed to be very close to the floor to detect small everyday object on the floor, and this limits the detection distance of object. If a low cost, small-sized LRF with sufficiently small laser diffusion is available, it could replace the current one.

## 4. Identification Using Laser Reflectivity

### 4.1. Background Subtraction and Clustering for Detection of Objects

A simple background subtraction and clustering method are applied to detect and localize unknown objects. To obtain background data, we remove all the movable objects from the room and measures range data of the room by the LRF The data are expressed in 2D range-angle space, namely the polar space. Over several scans, a set of observed distances is collected. Then the most frequently observed distances for each scan angle are used as the background data. At the measurement of object, the background data is subtracted from the scan data, and only foreground points are left. Next, we transform the data into 2D Cartesian space. Then, the data obtained by the direct measurement and the indirect measurement are handled in the same 2D coordinate frame (Figure 2a). Next, we cluster 2D points in a 2D coordinate frame using nearest neighbour(NN) algorithm. At first, the clustering is made separately for the data by direct measurement and for the data by indirect measurement. The distance tolerance threshold value for the NN-algorithm is set as *rθ* + *α* where r[mm] is distance from the LRF to each point, *θ*[rad] is the angle resolution of the LRF and *α* is the accuracy of the LRF. Finally, we merge clusters of the direct measurement and the indirect measurement if the minimal distance among them is less than 30 mm.

### 4.2. Normalization of Reflection Intensity and Identification of Object

**Figure 3.**
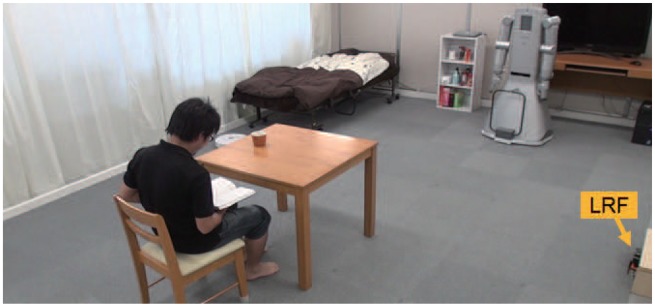
Objects in daily environment.

**Figure 4.**
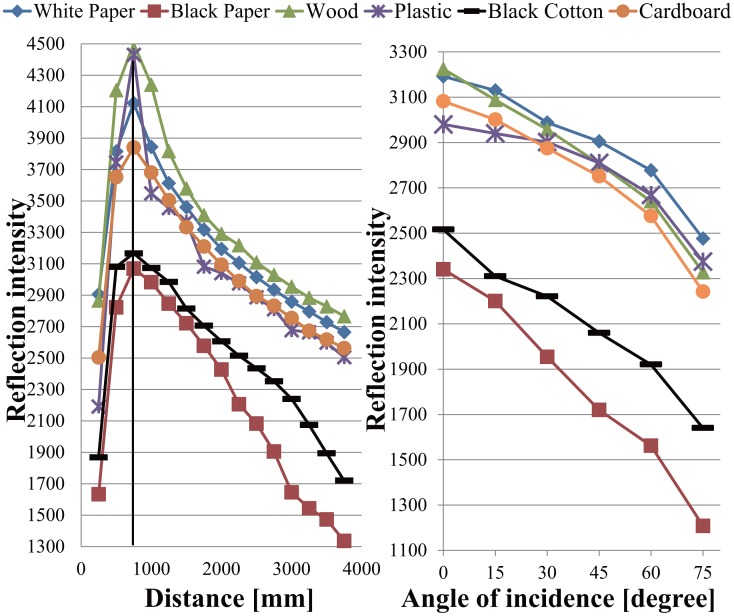
Experiment results of reflection intensity *vs.* distance and angle of incidence.

The LRF measures not only distance values but also intensity values of laser reflections. We use reflection intensity and position information to identify people, robots, furniture, and everyday objects made of wood, paper, plastic and rubber in a daily environment (Figure 3). Figure 4 shows the reflection feature for each object made of different material. The reflection intensity varies depending not only on the optical property of objects but also on the distance and angle of incidence of the laser beam against the object surface. Due to the hardware constraint of the LRF, the reflection intensity is not reliable when the object surface is located closer than 800mm as shown in Figure 4.

(1) $$\text{Intensity}=K_{d}I_{q}\frac{\cos{\left(\alpha \right)}^{0.196}}{r^{0.287}}$$

(2) $$\text{IntrinsicIntensity}=\text{Intensity}\frac{r^{0.287}}{\cos{\left(\alpha \right)}^{0.196}}$$

$$\begin{array}{lll} K_{d} & : & \text{diffuse reflection coefficient}\\ I_{q} & : & \text{the power of the light source}\\ \alpha & : & \text{angle of incidence on the surface}\\ r & : & \text{distance from the light source} \end{array}$$

Therefore, we used the reflection intensity only when the object surface is found far over the critical distance of 800 mm. Then, we obtained Equation (1) by curve fitting using the experiment data that satisfies the above mentioned condition. Finally, the approximate intrinsic intensity Equation (2) was obtained using Equation (1) with measured intensity, r and *α* from LRF.

**Figure 5.**
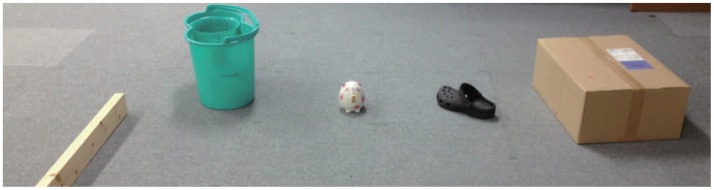
Experiment setups.

**Figure 6.**
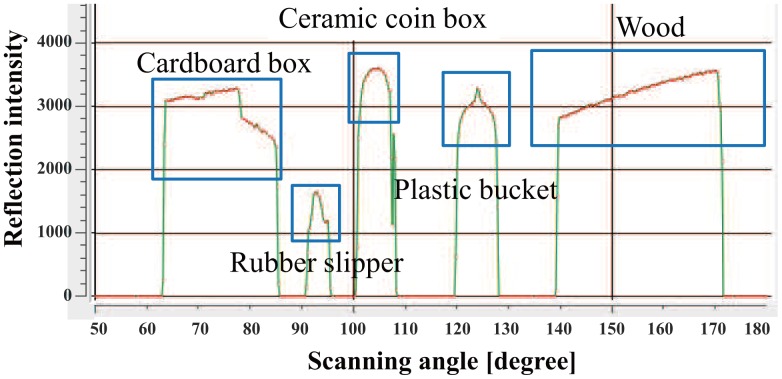
Reflection intensity value of each object.

**Figure 7.**
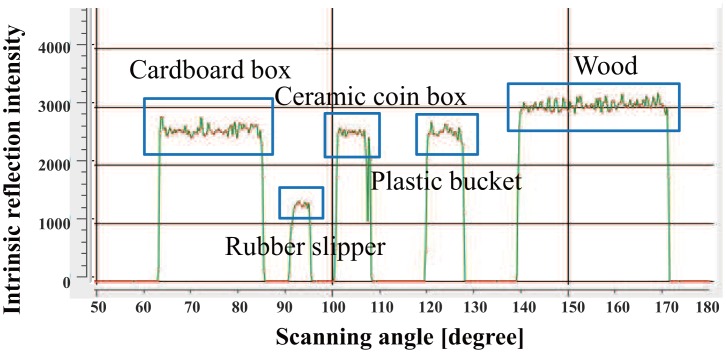
Normalized reflection intensity value of each object.

Next, we evaluate the effect of the normalization using Equations (1) and (2). A piece of wood, a red bucket, a green plastic bucket and a cardboard box were placed at 2–3 m in front of the LRF (Figure 5). The reflection intensity data from the surface of each object is shown in Figure 6. Moreover the normalized reflection intensity of each object is obtained in Figure 7. If there is a difference in the reflection intensity among the objects, then they can be identified immediately.

Identification by the normalized reflection could be improved if we use a high performance LRF having higher resolution of reflection intensity measurement with lower noise level. Actually, we have used a commercially available LRF since it is low cost and small-sized, and therefore suitable in the everyday environment.

## 5. Improvement of Detection and Position Measurement by Attaching Retroreflective Materials

### 5.1. Expanding Reflection by Attaching the Retroreflective Material

The measurable data corresponds only to a partial profile of object from the LRF Moreover some objects may be invisible due to the occlusion by other objects. In addition, it may often be difficult to separate the multiple clusters when they are closely located. This happens for example when a person approaches a table or sits down on a chair. Therefore, it is not easy to accurately identify and track objects in real time.

To solve these problems, we use the reflection intensity and position information obtained from the target surface. Although it is not possible to identify all objects by additionally using reflection values, it would be easier to identify unknown clusters if we could eliminate easily identifiable objects by reflectance.

**Figure 8.**
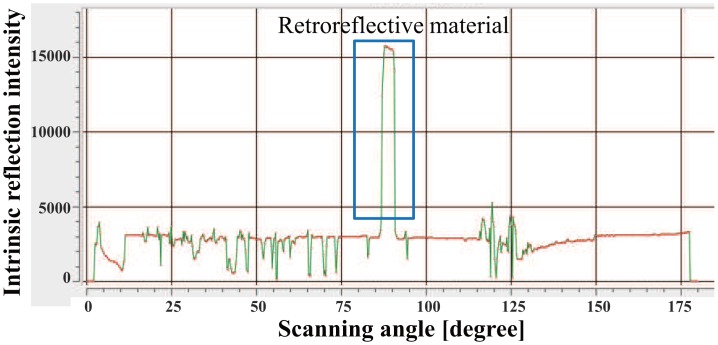
Reflection intensity value of retroreflective material.

If there is not enough difference in the reflection value of each object, we cannot identify the object using only reflection intensity. To solve this problem, we attached a tape made of retroreflective material to the object. Thus, the difference of the reflection features becomes larger (Figure 8). Using this simple method, we will be able to identify objects based on the distance and the reflection intensity.

### 5.2. Improvement of Reflectance Detection by Attaching Retroreflective Materials

**Figure 9.**
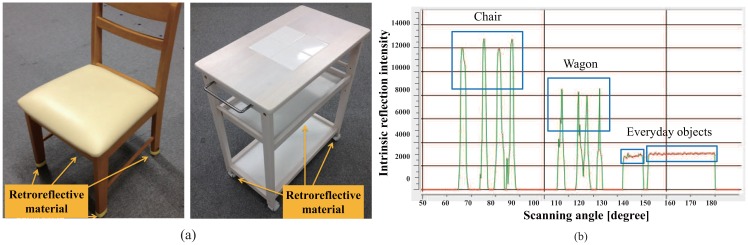
Attaching retroreflective materials and position measurement using reflected laser beam and diffused laser beam: (**a**) position of retroreflective material, (**b**) distinctly different intensity compared to the other objects.

Our daily life environment (Figure 3) contains some furniture that can be easily moved by a single person, for example, a chair and a dining wagon (Figure 9a). We attach the retroreflective material on the surface of legs of the chair and wheels of the dining wagon where the laser scans. Furthermore, we attach semi-transparent sheet over the retroreflective material to control the reflection intensity. As a result, they will be easily identified only by the reflection intensity as shown in Figure 9b.

Figure 9a shows how the retroreflective material is attached to the legs of movable furniture. Since the tags are small, they will not affect general appearance of these objects. This is good for keeping our daily environment as it used to be, even if service robots are working around.

### 5.3. Measurement of Robot Pose by Coded Reflection

It is not possible to determine the orientation of object based on the distance value obtained by LRF if the horizontal cross-section of the object is rotationally symmetric with respect to the vertical axis of rotation. There are certain objects that have circular cross-section shapes. A cylindrical mobile robot is a typical example. We developed a method to measure the orientation in such cases.

**Figure 10.**
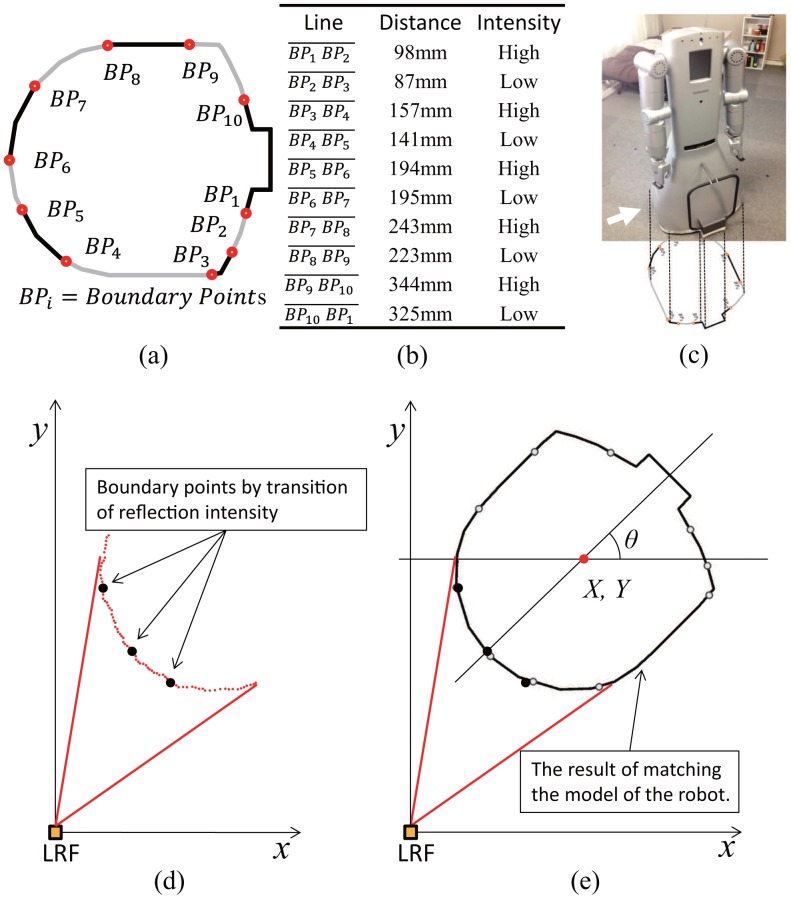
Example for measuring the robot's pose: (**a**) reflection feature, (**b**) features between points, (**c**) target location, (**d**) laser scan data, (**e**) result of matching.

The idea is to attach distinguishable optical features around the robot base. An optical feature in our case is a transition between reflective and not reflective material. These transitions are indicated in Figure 10a as boundary points BPs. Geometric distance between optical features is designed so that they are distinguished from each other around the robot base as shown in Figure 10a–c.

Once the robot base is scanned by the LRF, visible optical features are detected and identified (Figure 10d), and then matched with the robot base model (Figure 10e). The complete pose of the robot is computed based on the position of identified features.

## 6. Experiments

### 6.1. Reduction of Occlusion by Mirror

**Figure 11.**
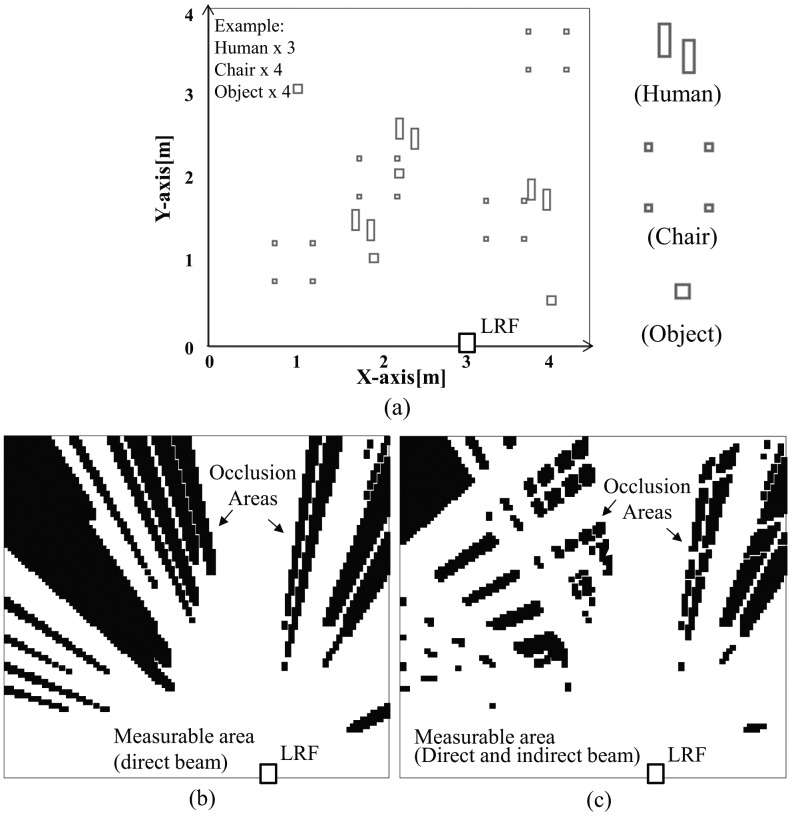
Simulation results of measurable area and occlusion areas: (**a**) position of human, chairs and objects, (**b**) measurable area and occlusion areas by direct laser beam, (**c**) measurable area and occlusion areas by direct laser beam and indirect laser beam.

The strip of mirror attached to the wall effectively reduces the occlusion area caused by objects. We performed simulation to quantitatively evaluate the reduction of this occlusion. Figure 11a shows an example of a simulated floor map of a room (4 m × 4 m). Obstacles are modeled by rectangles of different size: a human foot (0.25 m × 0.08 m), a leg of chair (0.05 m × 0.05 m), and an everyday object (0.1 m × 0.1 m). Figure 11b shows the occlusion area (black) and the measurable area (white) by direct laser beam of the LRF By using reflected laser beam, the occlusion area is reduced and the measurable area is expanded as shown in Figure 11c.

Various different indoor situations have been investigated: the number of people, pieces of furniture and objects on the floor is changed; thousands of different layouts are randomly generated for each set of objects.

Table 2 summarizes the result of the simulation. The first row of the Table 2 describes the case where a person and a chair are both on the floor. The total of cross section by a person and a chair is 0.05 m^2^. The layout of the human and the chair is changed randomly 1000 times. Then the average measurable area by the direct laser beam covers 94.5% of whole floor. Addition of the measurement by the reflected laser beam increases the area up to 99.2%. This means that 86.3% of occluded area is recovered to be measurable area.

As the number of people, pieces of furniture and objects on the floor increases, the measurable area by the direct laser beam decreases as shown in column 5 of Table 2. However, the decrease is slowed down when the reflected mirror is used as shown in column 6. The occluded area is effectively recovered by the mirror as shown in column 7. Thus we have confirmed the good performance of measurement with only one mirror.

### 6.2. Accuracy Evaluation for Position Measurement of Robot

**Figure 12.**
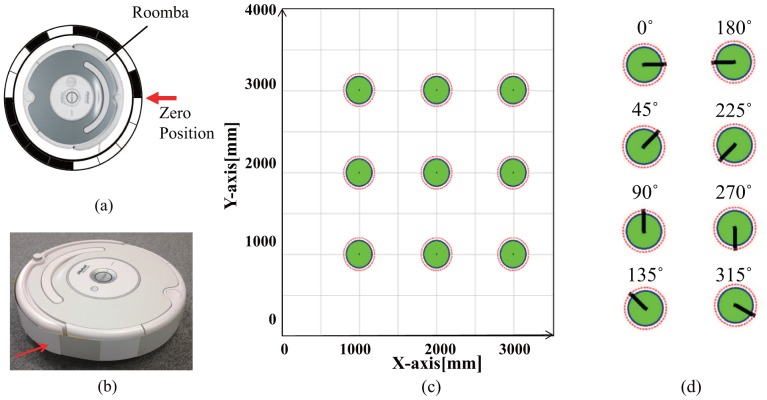
Evaluation of pose error: (**a**) reflection encoded tape, (**b**) target location, (**c**) evaluation of position error (x, y axis), (**d**) evaluation of direction error (*θ*).

We evaluate accuracy for position measurement of robots by a coded reflection method. We have wrapped a strip of reflection encoded tape(Figure 12a,b) around the body of a robot (Roomba, iRobot) and evaluated accuracy of the pose measurement. The robot was located at 9 different positions as shown in Figure 12c. At each position, the robot is replaced in 8 different orientations (Figure 12d). In the experiments, we used a regular grid marked on the floor. Then the robot was set by hand at the marked position on the grid. Setting error could be around 1mm since the shape of the robot is cylinder. We have measured 100 times for each pose and obtained mean errors of 5.6 mm along x-axis, 3.5 mm along y-axis, and 3.4 degrees about vertical axis. Their standard deviations are 4.3, 4.1, and 3.0, respectively.

**Figure 13.**
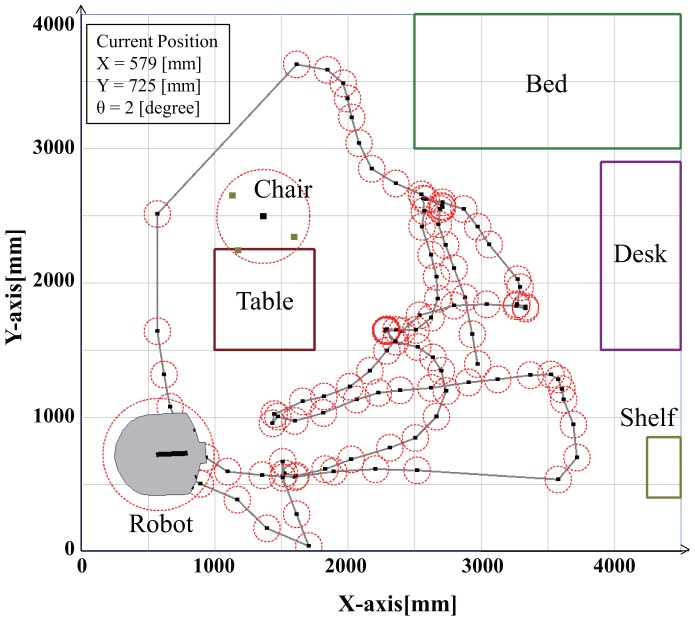
Trajectory of robot (5min).

We have performed other experiments for the measurement of robot trajectory by coded reflection method. In this experiments, we used a humanoid robot (SmartPal, Yaskawa Electric). Figure 13 shows the measurement result of the trajectory of the robot for 5 min. Since the occlusions occur between objects, position measurement failure occur 12 times in a total of 136 times.

### 6.3. Experiments for Measuring the Robot's Pose and Movable Furniture in Everyday Life Environment

**Figure 14.**
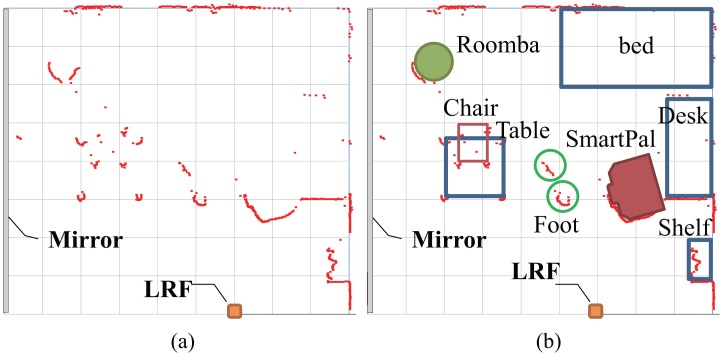
Original data by LRF and identification of objects: (**a**) original data points, (**b**) identification of objects.

We have performed experiments for measuring the position of robots, furniture, and people as shown in Figure 3. A bed is set in the corner of a room. A desk and a bookshelf are located along a wall. A table and a chair are positioned at the center area. Since the robots and the chair are equipped with retroreflective tape, they are immediately identified based on the reflectance intensity value by the LRF sensing system. Distance data of unmovable furniture are treated as background data. The original data obtained by the LRF is cluttered with many data points as shown in Figure 14a. The clusters belonging to the robots and the chair are recognized based on the reflection values as shown in Figure 14b, and the clusters of human feet are obtained in the remaining clusters.

## 7. Conclusions

In this paper we have presented a new measurement system of the pose of objects on the floor in the individual space for a service robot working in daily life environment. We have presented three ideas. (1) A floor sensing system using a LRF and a mirror; (2) Identification using laser reflectivity; (3) The improvement of detection and position measurement by attaching retroreflective materials.

We use a combination of both reflection intensity and position information obtained from the target surface by LRF. The proposed floor sensing system design enables the acquisition of the above mentioned information by using only one LRF and mirror. Since the area of sensing is limited to a scanning plane parallel to the floor surface and just a few centimeters above it, there may be less or smaller obstacles that intersects the sensing area in our target environment. This setting of the scanning plane together with the usage of the mirror effectively enables the LRF scanning to cover the whole room while mitigating occlusion by obstacles. Also, This system is very suitable for usage in daily life environment because of many advantages: it is very robust against illumination change; it has very low risk of invasion of privacy of residents since it does not capture full body image day and night whereas it is the case if the conventional camera is used.

The LRF may suffer from severe occlusion due to accidental positioning of an object close to the laser. However, such a situation can be minimized by carefully selecting the setting position of the LRF. Actually the LRF is set to the wall near the entrance door, since the resident usually does not place objects on the nearby surface of the entrance. In the case of accidental positioning of an object close to the laser, it is automatically detected. Thus we can design the system so that it calls a nurse or a robot to remove it. This may be constraint but can be acceptable because of much bigger advantage of the floor sensing system.

Method of measurement of objects and robot pose using the reflectance and distance data of the LRF have been implemented and the experiments validate effectiveness of our approach.

## Figures and Tables

**Table 1.** Diffuse height of laser beam with respect to distance and scanning angle.

**d[mm]**	**1000**	**2000**	**3000**	**4000**
h1[mm]	3	4	5	6
h2[mm]	4	24	50	62

**Table 2.** Simulation results of measurable range and recovery ratio.

**Total Size [m^2^]**	**Human**	**Chair**	**Object**	**Direct Method [%]**	**Combined Method [%]**	**Recovery Ratio[%]**
0.05	1	1	0	94.5	99.2	86.3
0.10	2	2	0	89.6	96.9	69.8
0.15	3	3	0	84.9	93.9	59.5
0.20	3	4	4	79.4	88.4	43.8

Average value				86.7	94.7	60.1
